# Green-Biased Technical Change and Its Influencing Factors of Agriculture Industry: Empirical Evidence at the Provincial Level in China

**DOI:** 10.3390/ijerph192316369

**Published:** 2022-12-06

**Authors:** Yan Wang, Lingling Zuo, Shujing Qian

**Affiliations:** School of Business, Ningbo University, Ningbo 315211, China

**Keywords:** agriculture, green-biased technical change, SBM model, Malmquist–Luenberger index

## Abstract

The continued expansion of agriculture must contend with the dual pressures of changing factor endowment structure and constrained resources and environments. The main purpose of this paper is to provide feasible ideas for high-quality agricultural development in the transition period through the research on the green-biased technical change in Chinese agriculture. This paper selects China’s provincial panel data of the agriculture industry from 1997 to 2017, combining the DEA-SBM model and Malmquist–Luenberger index decomposition method to calculate the green-biased technical change (BTC) index; second, the influence mechanism of BTC is empirically investigated by using the panel data regression analysis approach. The results show that: (1) in China’s agriculture industry, BTC is the driving force behind long-term and steady improvement of technological advancement. Specifically, input-biased technical change (IBTC) has a substantial enhancing effect on agricultural green total factor productivity (GTFP), whereas output-biased technical change (OBTC) has a certain inhibiting effect. (2) On the whole, the tendency of capital substituting for labor and land is very evident, whereas the biased advantage of desirable output is not particularly prominent. (3) The BTC index in Chinese agriculture varies regionally. The eastern region has the highest IBTC index but the lowest OBTC index. (4) The degree of marketization, urbanization, capital deepening, financial support for agriculture, and other factors have a promoting effect on IBTC, whereas most of them have a restraining effect on OBTC. There is evident regional heterogeneity in the effect of environmental regulation intensity on BTC. The following are the primary contributions of this paper: based on national conditions in China, this paper empirically explores the changes and internal rules of green-biased technical change in China’s agriculture industry from various regional viewpoints. It provides an empirical foundation for the regional diversification of agricultural green transformation.

## 1. Introduction

Since the reform and opening up, China’s agriculture has made outstanding progress. With less than 10% of the world’s land, China feeds more than 20% of the world’s population, effectively ensuring national food security and a steady supply of vital agricultural products [1]. However, the continued expansion of agriculture must contend with the dual pressures of changing factor endowment structure and constrained resources and environments. On the one hand, with the acceleration of industrialization and urbanization, the traditional agricultural factor supply, including cultivated land and labor force, has undergone significant changes [2,3]. On the other hand, a high environmental cost came along with this great success [4]. According to the Bulletin on the Second National Census of Pollution Sources, the national agricultural pollution discharge of chemical oxygen demand (CODcr), total nitrogen (TN), and total phosphorus (NP) in 2017 made up 49.77%, 46.52%, and 67.22% of all national emissions, respectively. Moreover, it is important to not undervalue the amount of carbon emissions produced by agricultural production processes such as rice farming and livestock raising [5,6]. At the 20th CPC National Congress, it was proposed to accelerate the green transformation of the development mode, and adhere to coordinated promotion of carbon reduction, pollution reduction, green expansion, and growth. The green development of agriculture is an important part of it, and how to resolve the “tripartite dilemma” of resources, environment, and development has become a practical problem that needs to be solved urgently.

For a very long time, China’s agricultural development and the expansion of the national economy have been greatly influenced by technical change. It is also an important means of bridging the gap between the demands for resources, the environment, and development. Even though the majority of neoclassical growth theories view technical change as exogenous and neutral, in most instances [7,8], the process of technical change and heterogeneity of factor endowment will result in different preferences for factor input, which will then result in different preferences for technical change [9,10,11]. Additionally, when technical change tends to conserve factor resources and reduce undesirable output, energy conservation and emission reduction can be achieved while promoting growth, reflecting the high-quality development objective of green production technology progress [12,13]. Then, a question arises: in the Chinese agriculture industry, what function does the green-biased technical change play in the agricultural growth process? Does it reflect the relative rarity of the factors, and does it successfully ease off the conflict between development and the environment? What are the driving forces behind the growth of green-biased technical change? Based on the research purpose above, this paper intends to measure the green-biased technical change by using the Chinese agriculture industry as a case study, analyze its contributions to agricultural growth, and investigate its bias characteristics. Additionally, this paper discusses the factors that influence green-biased technical change. The accomplishment of these research objectives will serve as a significant comparison value for the high-quality development of China’s agriculture during this phase of transition.

The marginal contribution of this study in comparison to earlier papers is as follows: first, this paper implements the DEA-SBM method to incorporate agricultural non-point source pollution and agricultural carbon emissions into the scope of the investigation of undesirable output. Based on China’s national circumstances, the pollution accounting method is revised in order to produce more accurate results and valuable policy references. Second, in contrast to the research on multi-factor input of agriculture industry, this paper only incorporated capital, land, and labor into a unified analysis framework through the accounting of provincial agricultural capital stock, so as to accurately grasp the changes and internal rules of green-biased technical change from a macro perspective. Thirdly, through the accounting, decomposition, and identification of green-biased technical change, as well as the regional investigation of the impact mechanism, this paper not only extends the existing research, but also offers an empirical foundation for the formulation of regional differentiation policy.

## 2. Literature Review

Since Acemoglu systematically discussed biased technical change [11,14,15,16], the biased technical change theory has gradually emerged as a fresh way to understand unbalanced economic development and growth. The Hayami and Ruttan (1971) theory of induced technological innovation in agriculture [10] has emerged as an important theoretical tool for assessing the progress of agricultural technology [17].

The parametric method and the non-parametric method are the two main solutions used to measure biased technical change. Stochastic frontier analysis (SFA) and the normalized supply-side system approach (NSS) are examples of parametric methods. Data envelopment analysis (DEA) is an example of a non-parametric method. The first strategy is called a normalized supply-side system (NSS). The most representative examples are two publications by Klump [18,19]. As research continues, academics apply the method to industries in order to analyze the bias of technical change [20,21,22]. Wang et al. (2015) employed this method to study the biased technical change in China’s agricultural sector [23]. The second technique is stochastic frontier analysis (SFA). Given the diversity of input factors, the CES function with fixed elasticity of substitution is rarely employed. In contrast, the translog production (cost) function with variable elasticity of substitution is gaining increasing attention [24,25,26]. The SFA method was employed in the field of agriculture by foreign researchers Taylor et al. (1986), Battese (1992), and Kalirajan (1996) to assess changes in total factor productivity [27,28,29]. Recent representative works on the bias of agricultural technical change in China include Kong et al. (2018) and Gong (2018) [2,17]. They both confirm the existence of biased technical change. The third method is data envelope analysis (DEA). To measure productivity, Caves et al. (1982) first suggested combining the DEA model and the Malmquist Index [30]. Later, Färe et al. (1994) and Färe et al. (1997) conducted additional research on this index and, from input and output, respectively, decomposed the biased technical change index [31,32]. The use of non-parametric techniques eliminates bias that results from the subjective setting of production functions and is appropriate for complex systems with numerous inputs and outputs [33,34]. Some scholars brought negative environmental output into the research framework and analyzed the biased technical change and its influential mechanism in different fields [12,33,35,36,37]. In the field of agriculture, Managi et al. (2004) and Singh et al. (2012), respectively, measured the biased technical change of agriculture in the United States and India [38,39]. The recent representative literature that applied the DEA method to study the biased technical change in Chinese agriculture includes the study of Yin et al. (2018) and Hu et al. (2021) [3,40]. However, neither the discussion of output-biased technical change based on agricultural pollution emission data nor its influence mechanism are included in these two studies.

Compared to the measurement of biased technical change, research on the influencing factors of biased technical change deserves equal attention. Generally speaking, the categories of scholars’ discussions are classified as follows: firstly, factor markets. Hicks (1932) noted that changes in the prices of production factors could encourage inventions, especially those that directly save relatively expensive factors [9]. Hayami and Ruttan (1971) explained the bias of technology generation and change under the given natural resource conditions, and empirically examined the differences in agricultural technology innovation and agricultural development paths between the United States and Japan [10]. Huang et al. (2020) and Liu et al. (2022) also empirically confirmed the viewpoints above [12,33]. Secondly, the policy. Gong (2018) studied the impact of a series of market-oriented fundamental reforms on biased technical change in agriculture since China’s reform and opening up [17]. Government innovation subsidies have a significant impact on biased technical change, according to an empirical study by Liu et al. (2022) [12]. The factor of environmental regulation has also become a hot topic of discussion in recent years. There are mainly two viewpoints: they are “compliance costs theory” [41] and “innovation compensation theory” [42], respectively. Some literature conducted empirical verification and found that the aforementioned two effects may exist in various circumstances [12,43,44,45,46]. The third is global trade. However, results on the impact of global trade on biased technical change are very different in specific empirical studies for various industries [12,24,33,47]. Fourth, the additional factors. Existing research has also examined the impact of property rights structure, industry size, energy consumption structure, R&D intensity, industrial structure, urbanization level, marketization degree, and foreign direct investment on biased technical change [12,24,33,46].

In conclusion, the current literature is valuable for future research on agricultural green technology progress. However, it still has drawbacks: (1) research on green-biased technical change in Chinese agriculture is not as common as it is in other industries. The difficulty in estimating provincial agricultural capital stock is one of the most important reasons. In most studies, intermediate inputs such as fertilizer, machinery, pesticides, agricultural film, water, and energy are substituted for agricultural capital factors [2,6,17,48,49,50,51]. However, these intermediate inputs are not ideal substitutes for agricultural capital factors [3,52]. (2) Most studies do not take into account agricultural undesirable output in sufficient amounts, which means that studies on agriculturally biased technical change lack full investigation of the important part of output bias. The following are the main reasons for inadequate consideration: first, the majority of the literature employs parametric methods that require strict assumptions on production functions, making it difficult to account for agricultural pollution emissions effectively. Second, to achieve the goal of high-quality development, both non-point source pollution and carbon emissions in agriculture cannot be ignored [51,53]. However, most current studies only consider one of them. Third, the measurement of agricultural carbon emissions in the majority of studies lacks breadth and precision. Some carbon emission coefficients are not based on the actual domestic situation and frequently copy foreign literature, and some studies fail to distinguish “carbon” and “carbon dioxide” accurately [5]. (3) Even though studies on the accounting of biased technical change in Chinese agriculture have been conducted, the influencing factors of biased technical change have not been empirically examined, and the varying influences on the different regions of China have not been considered.

## 3. Methods

### 3.1. Framework of Method Analysis

**Figure 1:**
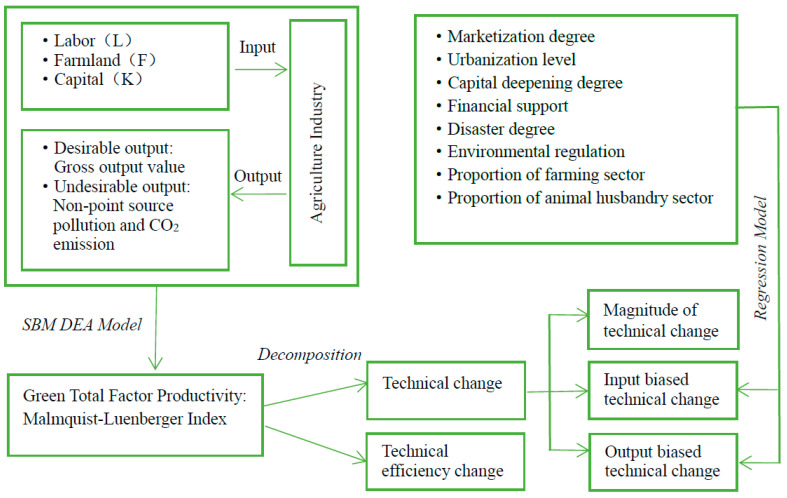
Analytical framework.

The research framework consists of three phases. First, this paper takes three factors of labor, land, and capital as the input ends, and takes desirable output and undesirable output (including agricultural non-point source pollution and agricultural carbon emissions) as the output ends to be included in the unified analysis framework of this research. Second, by configuring the DEA-SBM model, this paper constructs the Malmquist–Luenberger index, which can further decompose the biased technical change index. Following that, the bias identification method is used to pinpoint the input/output bias of technical change. Finally, regression analysis is used to identify the factors that influence biased technical change. Figure 1 depicts the analysis framework for this paper.

### 3.2. Calculation Method of Green-Biased Technical Change in Chinese Agriculture

#### 3.2.1. SBM Model Setting and Green Total Factor Productivity Decomposition

Tone (2001) proposed the SBM model to improve the traditional DEA model which failed to solve the problem with slack variables in the course of efficiency evaluation. Subsequently, Tone (2004) put forward the SBM model considering undesirable output. This model not only solved the slack improvement in efficiency evaluation, but also gave comprehensive consideration to the relationship between input, output, and bad pollution [54,55].

In the present study, each province in China is treated as a single decision-making unit (DMU) to construct the optimal production technology boundary. It is assumed that *K* province makes use of *N* input factors $X=\left(x_{1},\cdots ,x_{n}\right)$ to produce *M* desirable outputs $Y=\left(y_{1},\cdots ,y_{m}\right),y\in R_{m}^{+}$ and *I* undesirable outputs $B=(b_{1},\cdots ,b_{i}),b\in R_{i}^{+}$. When the input-output of the kth observed value in period *t* is expressed as $\left(x_{k}^{t},y_{k}^{t},b_{k}^{t}\right)$, the production possibility set containing undesirable output can be expressed as:

(1) $$p^{t}(x^{t})=\left\{\left(x_{k}^{t},y_{k}^{t},b_{k}^{t}\right)|:x_{n}^{t}\geq X\lambda ,y_{m}^{t}\leq Y\lambda ,b_{i}^{t}\geq B\lambda ,\lambda \geq 0\right\}$$

According to Equation (1), the SBM model considering undesirable output can be written as:

(2) $$\begin{array}{l} \rho^{*}=\min\frac{1-\frac{1}{N}\sum_{n=1}^{N}\frac{s_{n}^{x-}}{x_{nk}}}{1+\frac{1}{M+I}\left(\sum_{m=1}^{M}\frac{y_{m}}{y_{mk}}+\sum_{i=1}^{I}\frac{b_{i}}{b_{ik}}\right)}\\ s.t._{}{}_{}\;\;X\lambda +s^{x-}=x_{k}\\ \;Y\lambda -s^{y+}=y_{k}\\ \;B\lambda +s^{b-}=b_{k}\\ \;\lambda \geq 0,s^{x-},s^{y+},s^{b-}\geq 0 \end{array}$$

where $s^{x-},s^{y+},s^{b-}$ represent the slack values of input, desirable output, and undesirable output, respectively; and $x_{nk},y_{mk},b_{ik}$ refer to the nth input, the mth desirable output, and the ith undesirable output of the kth DMU, respectively. If and only if $\rho^{*}=1$, that is, when $s^{x-}=s^{y+}=s^{b-}=0$, the DMU is completely effective; otherwise, there is room for the improvement of both input and output.

Furthermore, inspired by the idea of Färe et al. (1994) and Chung et al. (1997), this paper starts by constructing the Malmquist–Luenberger index [31,56]. It is supposed that $\rho_{k}^{t}(x^{t},y^{t},b^{t})$ and $\rho_{k}^{t+1}(x^{t+1},y^{t+1},b^{t+1})$ are the efficiency values of the kth DMU at *t* and *t* + 1 period, respectively. Therefore, the agricultural green Malmquist–Luenberger index (*ML*) of each province is expressed as:

(3) $$ML_{k}^{t,t+1}={\left[\frac{\rho_{k}^{t}(x^{t+1},y^{t+1},b^{t+1})}{\rho_{k}^{t}(x^{t},y^{t},b^{t})}\times \frac{\rho_{k}^{t+1}(x^{t+1},y^{t+1},b^{t+1})}{\rho_{k}^{t+1}(x^{t},y^{t},b^{t})}\right]}^{\frac{1}{2}}$$

When $ML_{k}^{t,t+1}>1$, it indicates an increase in the green total factor productivity (GTFP) from period *t* to period *t +* 1. When $ML_{k}^{t,t+1}<1$, it indicates a decline in the GTFP from period *t* to period *t +* 1. According to the *ML* index decomposition method, it is further decomposed into two parts: technical change and efficiency change:

(4) $$\begin{array}{l} ML_{k}^{t,t+1}={\left[\frac{\rho_{k}^{t}(x^{t},y^{t},b^{t})}{\rho_{k}^{t+1}(x^{t},y^{t},b^{t})}\times \frac{\rho_{k}^{t}(x^{t+1},y^{t+1},b^{t+1})}{\rho_{k}^{t+1}(x^{t+1},y^{t+1},b^{t+1})}\right]}^{\frac{1}{2}}\times \frac{\rho_{k}^{t+1}(x^{t+1},y^{t+1},b^{t+1})}{\rho_{k}^{t}(x^{t},y^{t},b^{t})}\\ \begin{array}{ccc} & & = \end{array}TC_{k}^{t,t+1}\times EC_{k}^{t,t+1} \end{array}$$

where $TC_{k}^{t,t+1}$ represents the technical change of the kth DMU from *t* to *t +* 1 period, namely, the movement of technology frontier; and $EC_{k}^{t,t+1}$ denotes the change in relative efficiency, that is, the change in distance between the input–output combination and the production frontier.

To assess the bias of technical change, Färe et al. (1997), based on the study of Färe et al. (1994), decomposed the technical change (TC) index into the magnitude of technical change (MATC) and bias of technical change (BTC) index. Moreover, the BTC index can be further decomposed into the input-biased technical change index and output-biased technical change index [31,32]. Based on this, the specific decomposition process is expressed as follows:

(5) $$\begin{array}{l} TC_{k}^{t,t+1}={\left[\frac{\rho_{k}^{t}(x^{t},y^{t},b^{t})}{\rho_{k}^{t+1}(x^{t},y^{t},b^{t})}\times \frac{\rho_{k}^{t}(x^{t+1},y^{t+1},b^{t+1})}{\rho_{k}^{t+1}(x^{t+1},y^{t+1},b^{t+1})}\right]}^{\frac{1}{2}}\\ \begin{array}{ccc} & & = \end{array}\frac{\rho_{k}^{t}(x^{t+1},y^{t+1},b^{t+1})}{\rho_{k}^{t+1}(x^{t+1},y^{t+1},b^{t+1})}\times {\left[\frac{\rho_{k}^{t}(x^{t},y^{t},b^{t})}{\rho_{k}^{t+1}(x^{t},y^{t},b^{t})}\times \frac{\rho_{k}^{t+1}(x^{t+1},y^{t+1},b^{t+1})}{\rho_{k}^{t}(x^{t+1},y^{t+1},b^{t+1})}\right]}^{\frac{1}{2}}\\ \begin{array}{ccc} & & = \end{array}MATC_{k}^{t,t+1}\times BTC_{k}^{t,t+1} \end{array}$$

(6) $$\begin{array}{lll} BTC_{k}^{t,t+1} & = & {\left[\frac{\rho_{k}^{t}(x^{t},y^{t},b^{t})}{\rho_{k}^{t+1}(x^{t},y^{t},b^{t})}\times \frac{\rho_{k}^{t+1}(x^{t+1},y^{t+1},b^{t+1})}{\rho_{k}^{t}(x^{t+1},y^{t+1},b^{t+1})}\right]}^{\frac{1}{2}}\\ & = & {\left[\frac{\rho_{k}^{t+1}(x^{t},y^{t},b^{t})}{\rho_{k}^{t}(x^{t},y^{t},b^{t})}\times \frac{\rho_{k}^{t}(x^{t+1},y^{t},b^{t})}{\rho_{k}^{t+1}(x^{t+1},y^{t},b^{t})}\right]}^{\frac{1}{2}}\times \\ & & {\left[\frac{\rho_{k}^{t}(x^{t+1},y^{t+1},b^{t+1})}{\rho_{k}^{t+1}(x^{t+1},y^{t+1},b^{t+1})}\times \frac{\rho_{k}^{t+1}(x^{t+1},y^{t},b^{t})}{\rho_{k}^{t}(x^{t+1},y^{t},b^{t})}\right]}^{\frac{1}{2}}\\ & = & IBTC_{k}^{t,t+1}\times OBTC_{k}^{t,t+1} \end{array}$$

where MATC represents neutral technical change, which is used to measure the shift of production frontier; BTC indicates the biased technical change, which is used to measure the “non-neutral” transfer of technological frontier; IBTC refers to input-biased technical change, which is used to measure the change in marginal rate of substitution of different input factors caused by technical change; and OBTC denotes output-biased technical change, which is used to measure the promoting effect of technological progress on different proportions of various outputs in the case of multiple outputs. If IBTC>1(<1), it indicates that the input-biased technical change promotes (hinders) the enhancement of GTFP. Likewise, when OBTC>1(<1), it implies that the output-biased technical change promotes (hinders) the enhancement of GTFP. Additionally, when IBTC=1 and OBTC=1, it suggests that the technical change is Hicks-neutral.

#### 3.2.2. Identification Method of Green Technical Change Bias

The indexes IBTC and OBTC are used to measure the biased technical change from the perspective of input and output, respectively. However, it is difficult to know the specific bias of the input and output side by using the indexes alone. Weber and Domazlicky (1999) proposed a method, which combined biased technical change index and intertemporal variation of marginal substitution rate of factors, to analyze the bias of specific factors [57]. Based on the above arguments, and the idea of Huang et al. (2020) and Ding et al. (2020) [33,46], the specific method of identifying input bias of technical change is constructed as follows:

(7) $$\pi_{i,j}=\left(\frac{I^{t+1}}{J^{t+1}}/\frac{I^{t}}{J^{t}}-1\right)\times \left(IBTC-1\right)$$

It is assumed that there is technical change occurring from the *t* to *t +* 1 period. $\frac{I^{t+1}}{J^{t+1}}/\frac{I^{t}}{J^{t}}$ represents the ratio of the marginal substitution rate of factors I and J from *t* to *t* + 1, which indicates the change of factor allocation. At a time when IBTC>1 and $\frac{I^{t+1}}{J^{t+1}}/\frac{I^{t}}{J^{t}}>1$, $\pi_{i,j}>0$, the biased technological progress results from the relatively more inputs of factor I, which is referred to as factor I-driven technological progress (or factor J-saving technological progress). Conversely, when IBTC<1 and $\frac{I^{t+1}}{J^{t+1}}/\frac{I^{t}}{J^{t}}>1$, $\pi_{i,j}<0$, the large input of factor I contributes to technological retrogression. That is to say, technological progress is biased towards factor J, namely, factor J-driven technological progress.

Similar to the analysis of output-biased technical change, the output bias index $\pi_{YB}$ is expressed as follows:

(8) $$\pi_{Y,B}=\left(\frac{Y^{t+1}}{B^{t+1}}/\frac{Y^{t}}{B^{t}}-1\right)\times \left(OBTC-1\right)$$

where $\frac{Y^{t+1}}{B^{t+1}}/\frac{Y^{t}}{B^{t}}$ represents the ratio of the marginal substitution rate of the desirable output *Y* and the undesirable output *B* between the two periods. When $\pi_{Y,B}>0$, it indicates that technological progress is biased towards producing more desirable output while reducing undesirable output, which is referred to as environmentally friendly technological progress. In addition, the opposite is true for environmental degradation technological progress.

Finally, the methods used to identify the input and output biases of green technical change are summarized, as shown in Table 1:

### 3.3. Regression Analysis of Factors Affecting Green-Biased Technical Change

To further explain the differences in green-biased technical change among regions, this paper takes IBTC and OBTC as the explained variables to analyze the effects of various factors on biased technical change. The model is presented as follows:

(9) $$\begin{array}{ll} \left\{\ln IBTC_{it},\ln OBTC_{it}\right\} & =\alpha +\beta_{1}\ln MA_{it}+\beta_{2}\ln UL_{it}+\beta_{3}\ln CD_{it}\\ & +\beta_{4}\ln FS_{it}+\beta_{5}ER_{it}+\beta_{6}ER_{{}_{it}}^{2}+\beta_{7}\ln DR_{it}\\ & +\beta_{8}\ln PF_{it}+\beta_{9}\ln AH_{it}+\eta_{i}+\mu_{t}+\varepsilon_{it} \end{array}$$

where *i* and *t* represent the province and time, respectively; IBTC and OBTC denote the agricultural input-biased technical change and output-biased technical change of each province, respectively; and η, μ, and ε refer to the individual effect reflecting the regional differences of each province, the time effect changing over time, and other interference terms, respectively. The meaning of each explanatory variable is detailed as follows:

(1) Degree of marketization (*MA*). The degree of marketization is regarded as a significant index used to indicate the marketization mobility of agricultural products and agricultural factors [33]. Herein, Fan Gang’s marketization index is adopted to measure the depth and breadth of marketization reform carried out in each province.
(2) Urbanization level (*UL*). Urbanization is coupled with the transfer of substantial agricultural surplus labor and the non-agricultural process of arable land [33,58]. Herein, the proportion of urban population to the total population is used to measure the urbanization level.
(3) Agricultural capital deepening (*CD*). Capital accumulation and its deepening provide a crucial driving force for agricultural growth. According to the study of Li et al. (2014) and that of Kong et al. (2018) [2,58], there are three indicators selected: total power of agricultural machinery/10,000 people, fertilizer application amount (net)/1000 hectares, and effective irrigation area/1000 hectares. Then, the entropy method is applied to calculate the variables used to characterize the degree of agricultural capital deepening.
(4) Financial support for agriculture (*FS*). The financial support offered for agriculture reflects, to a large extent, the shift in the agricultural support policy enforced by the government and the actual strength of the agricultural public investment [5,52]. Herein, this paper uses the proportion of agricultural expenditure in total fiscal expenditure to measure it.
(5) Environmental regulation intensity (*ER*). It represents the trade-off made by the government between economic output and green development. Based on the research of Tian et al. (2022), this paper uses the proportion of investment in environmental pollution control in regional GDP to measure the intensity of environmental regulation [5]. In the meantime, to overcome the data missing in individual years of investment in environmental pollution control, the missing data are estimated by using the average proportion of the investment completed in anti-industrial pollution projects to the investment in environmental pollution control over the years. In addition, in order to study the nonlinear impact of environmental regulation intensity on biased technical change, the quadratic term of this variable is also included in the model.
(6) Agricultural disaster rate (*DR*). Given the special attributes of intertwined natural reproduction and economic reproduction for agriculture industry, consideration is given in this study to the impact of uncontrollable natural factors, such as agricultural disasters on the bias of technical change, which is based on the reference made to the study of Liu et al. (2021) [48]. In this paper, the agricultural disaster rate is measured by the proportion of agricultural disaster area to total sown area.
(7) Structure of agricultural sector. Allowing for farming and animal husbandry as the two major sectors of agricultural productions, as well as the significant contributors to non-point environmental pollution and carbon emissions [5,59], the impact of different sectors within agriculture on the green-biased technical change is investigated by using two indicators. They are the ratio/share of the two segments: farming (*PF*) and animal husbandry (*AH*), respectively.

## 4. Variable Definition and Data Sources

Given the availability of data, the consistency of statistical caliber, and the comparability made to various accounting indicators, this paper selects the period from 1996 to 2017 as the research time span (uniformly deflating the output value indicators at 1978 constant prices). On this basis, the provincial panel data in mainland China (excluding Hong Kong, Macao, and Taiwan) is used to analyze the agricultural, green-biased technical change and its influencing factors. Additionally, allowing for the traditional regional division and regional economic development in China, the sample provinces are categorized into three major regions: eastern, central, and western regions. Specifically, the eastern region covers 11 provinces: Beijing, Tianjin, Hebei, Liaoning, Shanghai, Jiangsu, Zhejiang, Fujian, Shandong, Guangdong, and Hainan; the central region comprises 8 provinces: Shanxi, Henan, Anhui, Hubei, Jiangxi, Hunan, Jilin, and Heilongjiang; and the western region consists of 12 provinces, Inner Mongolia, Guangxi, Chongqing, Sichuan, Guizhou, Yunnan, Shaanxi, Gansu, Qinghai, Ningxia, Xinjiang, and Tibet. The data of Chongqing are incorporated into Sichuan in the actual analysis, whereas Tibet is excluded from the sample due to the particularity of resource endowment conditions and data availability. Finally, the panel data of 29 provinces (including autonomous regions and municipalities) from 1996 to 2017 are gathered.

There are extensive data sources used in this study, mainly including The China Statistical Yearbook, China Rural Statistical Yearbook, China Agricultural Statistics 1949–2019, Provincial-level Statistical Yearbooks, and National Bureau of Statistics. These indicators and data sources are detailed as follows:

(1) Input factors. Three input factors, namely, labor (L), farmland (F), and capital (K), are adopted. Among them, the labor input is represented by the number of employees in the first industry at the end of the year; the farmland input is indicated by the sown area of crops, considering the multiple cropping index; the capital input is based on the treatment method proposed in the study of Li et al. (2014) and Feenstra et al. (2015) [58,60], so as to estimate agricultural capital stock (at constant prices in 1978) through the perpetual inventory method. The key data of agricultural capital stock accounting are sourced from Historical Data of China’s GDP Accounting and Statistical Yearbook of China’s Fixed Assets Investment. Additionally, the sample data used in this study are only available until 2017 because provincial fixed asset investment statistics have ceased to be published since 2017.
(2) Output. Desirable output (Y) is defined as the gross output value of farming, forestry, animal husbandry, and fishery (constant price in 1978). With regard to undesirable output (B), there are two pollution sources adopted: agricultural carbon emission (CO_2_) and agricultural non-point source pollution (ANSP, including chemical oxygen demand (CODcr), total nitrogen (TN), total phosphorus (TP) emissions). Moreover, the entropy method is applied to obtain the comprehensive index of agricultural environmental pollution.
(3) Agricultural carbon emissions (CO_2_) estimation and data sources. This paper takes into account a number of research results [5,6,61,62], with four aspects (agricultural materials, livestock breeding, rice cultivation, and agricultural energy) selected to measure the sources of agricultural carbon emission. Table 2 lists the specific reference sources of emission coefficient. The first category, the carbon emissions from agricultural materials, includes carbon emissions from the production and subsequent utilization of chemical fertilizers, pesticides, and agricultural films. As chemical fertilizer is an important contributor to China’s agricultural carbon emissions, its accounting results will directly affect the accuracy of the total agricultural carbon emissions. Therefore, unlike previous studies, this paper subdivides chemical fertilizers into nitrogen, phosphorus, potassium, and compound fertilizers, and uses carbon emission coefficients of different chemical fertilizer varieties that reflect China’s actual conditions to calculate them, respectively [63]. The second category is the carbon emissions from rice planting, with the dual differences of rice planting cycle and region taken into account for the choice over the rice carbon emission coefficient. The third category, the carbon emissions from livestock and poultry breeding, includes CH_4_ and N_2_O emissions from livestock and poultry intestinal fermentation and excreta. This is purposed mainly to investigate cattle (including beef cattle, dairy cattle, buffalo), sheep (including goats and sheep), pigs, poultry, and other major livestock and poultry varieties. The fourth category, agricultural energy carbon emissions, involves agricultural diesel oil as a major contributor. Therefore, the calculation formula for agricultural carbon emission is expressed as follows:

(10) $$C=\sum C_{c}=\sum T_{c}\delta_{c}$$

where *C* and *C_c_* (subscript indicates the category of carbon sources) represent the total amount of agricultural carbon emissions and the carbon emissions caused by various specific carbon sources, respectively; *T_c_* and $\delta_{c}$ denote the actual number of various types of carbon sources and their corresponding carbon emission coefficient, respectively. Finally, the calculated greenhouse gases are converted into standard CO_2_. According to the IPCC Fourth Assessment Report, the CO_2_ conversion coefficients are 44/12, 25, and 298 for *C*, CH_4_, and N_2_O, respectively.

(4) Agricultural non-point source pollution (*ANSP*) estimation and data sources. The unit analysis method is developed to investigate and calculate agricultural non-point source pollution, including chemical oxygen demand (CODcr), total nitrogen (TN), and total phosphorus (TP). According to the studies of Lai et al. (2004), Chen et al. (2006), and Chen et al. (2021) [49,66,67], the following formula is used to calculate the emissions of *ANSP*:
  (11) $$ANSP=\sum EU_{activity}=\sum \sum EU_{class}=\sum \sum \sum E_{Unit}\times EUA$$

*ANSP* refers to the non-point source pollution emission, and $EU_{activity}$ represents the agricultural production activities that lead to non-point source pollution. $EU_{\mathrm{class}}$ indicates the type of pollutants, $EU_{\mathrm{unit}}$ denotes the non-point source pollution unit, and EUA represents the single unit pollution discharge. The method used to calculate EUA is expressed as follows:

(12) $$EUA=\sum_{i}EU_{i}\rho_{ij}(1-\eta_{i})C_{ij}(EU_{ij},S)=\sum_{i}PE_{ij}\rho_{ij}(1-\eta_{i})C_{ij}(EU_{ij},S)$$

where $EU_{i}$ represents the statistic of unit *i*; $\rho_{ij}$ indicates the pollutant production intensity coefficient of unit *i*; $\eta_{i}$ refers to the coefficient that indicates the utilization efficiency of related resources; $PE_{ij}$ denotes the production of non-point source pollution; and $C_{ij}$ stands for the emission coefficient of pollutant *j* of unit *i*, as determined mainly by unit and spatial characteristics (*S*). Table 3 lists the agricultural non-point source pollution units in China. For the emission coefficients of pollutants, they are based mainly on the reference made to the study of Lai et al. (2004), Chen et al. (2021), and the Bulletin on the First National Census of Pollution Sources [49,66].

## 5. Empirical Results

### 5.1. Analysis of Green-Biased Technical Change in Chinese Agriculture

**Figure 2:**
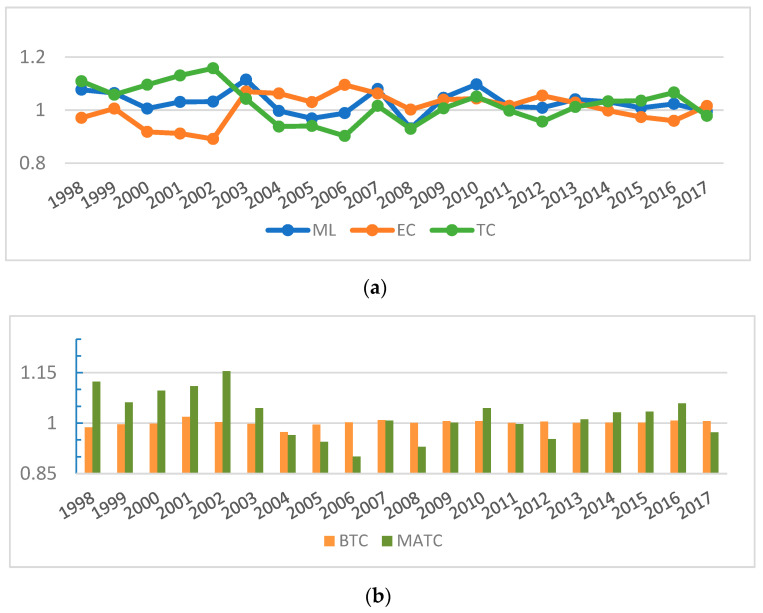
(**a**) Agricultural GTFP index and its decomposition; (**b**) TC index and its decomposition.

The geometric mean of the ML index was found to be slightly higher than 1 for the 29 provinces during the period from 1998 to 2017, which indicates an overall upward trend in agricultural green total factor productivity (GTFP). With regard to the composition of ML index of GTFP, stage characteristics are exhibited by the contribution of efficiency index (EC) and technical change index (TC) to its growth. As shown in Figure 2a, prior to 2003, the contribution of TC to ML growth was more significant compared to EC. During the decade from 2003 to 2013, however, the TC index declined, and EC played a more significant role in driving the increase of the ML index. In the years after 2013, the main contributor of ML index growth was gradually converted to TC. Overall, the geometric mean of the TC index is higher compared to EC, which means that the TC index is essential for promoting agricultural GTFP growth. Furthermore, the TC index can be decomposed into bias of technical change (BTC) and magnitude of technical change (MATC) indexes. As shown in Figure 2b, although the MATC index made great contributions to the TC index in some years, it continued to decline and fluctuated sharply since 2003, which is the main reason why the TC index experienced a 10-year downturn. For a long time, there was a relative stability maintained in the contribution of the BTC index to the TC index, and it reaches above 1 in most years. Especially since 2003, it has played an increasingly prominent role in maintaining the stability of the TC index. To sum up, biased technical change is crucial to sustaining the enhancement of agricultural green total factor productivity in the long run.

Table 4 lists the results of BTC index decomposition for different regions. Specifically, the average value of the OBTC index is slightly lower than 1 in the three regions, indicating the room for further improvement of the OBTC index. From the regional differences of the OBTC index, the improvement in the western and central regions is more significant than in the eastern region over time. Except for the central region, the IBTC index is almost greater than 1. That is to say, it exerts a positive effect on GTFP as a whole, and the advantage in the IBTC index is the most significant in the eastern region.

**Figure 3:**
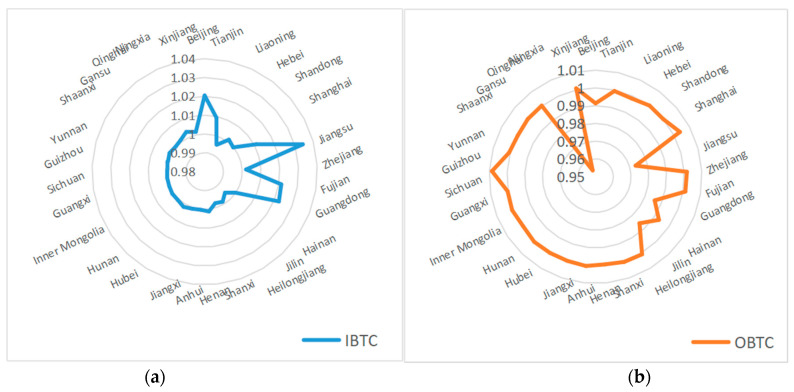
(**a**) Statistics of IBTC indexes of each province; (**b**) statistics of OBTC indexes of each province.

Figure 3 shows the statistics of the IBTC and OBTC indexes for each province. According to the radar chart of the IBTC index, the average IBTC index exceeds 1 in most provinces, and the most prominent input-biased technological progress is achieved mainly in those provinces located in the eastern region, including Beijing, Tianjin, Jiangsu, Fujian, Guangdong, and so on. Comparatively, the OBTC index is less than optimistic, basically around “1”, with the radar map showing a more obvious regional “collapse” in some eastern provinces. Notably, despite an excellent performance in the IBTC index in the eastern region, there are also some downsides of the OBTC index (such as Guangdong and Jiangsu), which has, to some extent, offset the growth of GTFP brought about by input-biased technological progress.

### 5.2. Bias Identification Analysis of Green Technical Change in Chinese Agriculture

**Figure 4:**
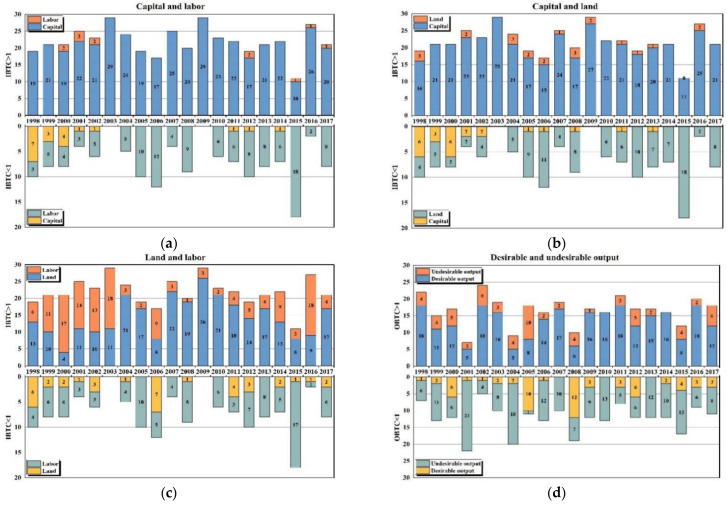
Statistics of input bias and output bias: (**a**) capital–labor combination; (**b**) capital–land combination; (**c**) land–labor combination; (**d**) desirable–undesirable combination.

Figure 4 shows the classified statistical results of agricultural input bias and output bias during the period from 1998 to 2017. First of all, the factor combinations of capital-labor and capital-land both show a relatively consistent trend; that is, China’s agricultural production tends to use more capital while saving labor and land factors. As for the input combination of labor-land factors, land use bias is slightly more than labor use bias. Secondly, according to the trend of contribution made by input bias to technical change (the statistical results of the classification of whether the IBTC index exceeds 1 in Figure 4), there is a similar regularity, whether it is a combination of capital-labor factors or of capital-land factors. That is to say, in provinces with IBTC > 1, the trend of capital substituting for labor and land is obvious, especially noticeable in the years after 2003. It indicates the promoting effect of capital-biased technical change on the improvement of GTFP. This conclusion is consistent with the theory of induced technological innovation [10], and coherent with the findings of Yin et al. (2018) [3]. In contrast, the input bias of labor or land factor is reflected more as technical regress (IBTC < 1), which, to some extent, reduces GTFP. Regarding the combination of land-labor factors, there is a certain trend of land replacing labor factor when IBTC > 1. Similarly, labor is more likely to substitute for land factor when IBTC < 1. This trend is more evident in the years after 2003. However, this trend is less stable compared with the capital–labor and capital–land factor combinations.

According to the output bias statistics, the desirable output bias of agricultural production is slightly more than the undesirable output bias in the provinces during the period from 1998 to 2017. Moreover, as for the contribution of output bias to technological progress, there is a certain trend of desirable output substituting for undesirable output on the whole when OBTC > 1. However, the opposite is true when OBTC < 1. It is suggested that green production technology exerts a positive effect on GTFP. By taking into account the finding that the previously calculated average of the OBTC index is less than 1, it can be found out that the promoting effect of agricultural green production technology remains insufficient, which is a major constraint on the improvement of GTFP. Therefore, it is still worth paying adequate attention to the environmental pollution caused by agricultural production. However, it is also worth noting that the trend of desirable output substituting for undesirable output has started to show a positive sign since 2008, especially in the years after 2015. One of the primary reasons for this change in recent years is that the state government has placed a significant emphasis on agricultural energy conservation and emission reduction. The specific calculation results indicate that, since 2015, major pollution emissions such as agricultural carbon emissions and non-point source pollution have all decreased to varying degrees, with the pollution of agricultural materials and livestock and poultry decreasing the most. The former is mainly due to the “zero increase in the use of chemical fertilizers and pesticides” launched by the Ministry of Agriculture and Rural Affairs in 2015. The latter is mainly due to the “2015 pollution reduction target” set in the 12th Five-Year Plan for the prevention and control of pollution from livestock and poultry breeding.

Table 5 lists the statistical results of input and output bias for various regions. Regarding the capital–labor and capital–land combinations, the eastern, central, and western regions show a clear trend of capital substituting for labor and land. As for the land–labor factor combination, the trend of land substituting for labor is most significant in the western region. According to the statistical results of output bias, the substitution effect of the desirable output on the undesirable output of the national total sample is slightly stronger. As suggested by the results of the regional comparison, the environmental problems caused by agricultural production are most severe in the eastern region, which reaffirms the previous analytical results.

### 5.3. Analysis of Influencing Factors on Green-Biased Technical Change in Chinese Agriculture

According to previous findings, the agricultural production in China shows an evident trend of capital substituting for labor and land. In addition to promoting IBTC, it also stimulates the growth of agricultural GTFP. However, the capital-driven application of agricultural technology has also caused environmental pollution problems at the output end. To some extent, it offsets the contribution of BTC to the growth of agricultural GTFP. Therefore, it is necessary to further analyze the influencing factors of green-biased technical change in Chinese agriculture, so as to explore the source of its growth.

Table 6 reports the descriptive statistics of main variables. According to the standard deviation, the variables lnDR and lnCD have the largest volatility, whereas the variables ER and ER^2^ register the smallest volatility. The skewness values indicate that four variables are skewed to the right and seven variables are skewed to the left. Seven variables have leptokurtic distributions because their kurtosis values exceed the threshold of 3. In contrast, four variables have platykurtic distributions because their kurtosis value fall below this threshold. Moreover, since all Jarque–Bera test values pass the significance test, the null hypothesis of normal distribution is rejected. Therefore, it indicates that the variables selected are non-normally distributed.

Before running any panel model on these data, a correlation matrix is computed to inspect the correlations between all variables selected by this paper, as shown in Table 7:

According to the results displayed in Table 7, the correlation coefficient ranges from −0.4871 to 0.7102. The results regarding the explanatory variables show that they are not highly correlated and, therefore, there is no multicollinearity risk. Since none of the correlations exceed the threshold of 0.8, it indicates that multicollinearity would not bias the following econometric estimations [68,69,70]. Furthermore, variance inflation factor (VIF) is adopted as an additional method to test multicollinearity risk. Since the maximum VIF value is 2.54, which is much smaller than 5, the risk of multicollinearity can be excluded [68]. The coefficient covariance matrix is also computed as follows (Table 8):

Table 9 lists the LLC and Fisher tests (including Fisher-ADF and Fisher PP) for the panel series as unit root tests to ensure the validity of the estimates. According to the results, each variable has passed the unit root test. It suggests the stability of the panel data in time series and its applicability for regression analysis.

Herein, IBTC and OBTC are treated as explained variables, respectively, so as to explore the differences in influencing factors among different regions through grouping regression. The estimations are tested across the pooled OLS model, fixed effects model, and random effects model. To estimate, the considered regression models were taken after analyzing the results of the F test, the Breusch–Pagan LM test, and the Hausman test. A summary of these test results are displayed as follows (Table 10):

According to the Table 10, the results indicate that the *p* value of Hausman test is established to be less than 10% level of significance. Therefore, the null hypothesis that the effects are random cannot be accepted. Additionally, the probability value of the F test, as well as the Breusch–Pagan LM test, is found to have less than a 10% level of significance. Hence, the fixed effects model is the most appropriate when testing the factors that influenced green-biased technical change.

Columns (1), (2), (3), and (4) of Table 11 show the regression results obtained for the influencing factors in IBTC across the country, eastern, central, and western regions, respectively. Columns (5), (6), (7), and (8) show the regression results obtained for the influencing factors in OBTC across the country, eastern, central, and western regions, respectively. Considering that the perturbation term of the econometric model may exhibit serial correlation, heteroscedasticity, or self-correlation, and that the number of cross-sectional units in this paper is larger than the time span, the standard error will be underestimated if the usual panel data estimation method is used; therefore, the Driscoll–Kraay standard error is used to correct for this [71,72].

Although the degree of marketization is insignificant in the national sample regression, it exerts a significant positive effect on the IBTC in the eastern region and a positive effect on the OBTC in the central region. The fiscal support offered for agriculture has the most significant effect on IBTC in the eastern region, whereas the impact on OBTC fails the significance test. The level of urbanization exerts not only a significant positive effect on IBTC in the whole country and the western region, but also a significant negative effect on OBTC in the eastern and central regions. This is because the rapid development of urbanization in the eastern and central regions leads to a decline in the available arable land area and the structure of consumption demand for agricultural products changes due to a fast-paced increase of urban population. To ensure the prompt supply of “rice bags” and “vegetable baskets”, it is inevitable for pollution emissions to increase because of being output-oriented, which impedes the progress of OBTC. In the eastern region, capital deepening exerts a significant positive effect on IBTC. According to the results of previous input-biased identification analysis, the factor market develops well in the eastern region, and the substitution of capital for labor and land factors is effectively promoted by the accumulation and deepening of agricultural capital. This is beneficial in promoting agricultural technological progress [3,58]. Additionally, capital deepening has a significant negative effect on OBTC in the eastern and central regions, but this effect is found positive in the western region. This means that the capital accumulation in the eastern and central regions makes the agricultural technology promotion and production management model still deviate from the goals of environment-friendly development. The disaster rate shows a positive effect to IBTC in the eastern region. The possible reason is that the agricultural capital in the eastern region is highly deepened, and the production can be rapidly adjusted even in the face of natural disasters. However, the disaster rate exerts a significant negative effect on the OBTC in the eastern region, indicating that natural disasters have lowered output expectations and further suppressed the promotion of agricultural green technology. The farming proportion and animal husbandry proportion both show a significant negative effect to the regression of the national total sample and cause a significant positive effect on IBTC only in the central region. This is because most provinces in the central region are the main grain producing areas, as well as the areas of highly developed animal husbandry. Moreover, there are remarkable results achieved in the scale and intensive management of planting and animal husbandry, which not only improves the marginal substitution rate of dominant factors, but also promotes technological progress [3]. However, the two variables make no significant contribution to OBTC in general. In particular, animal husbandry, which is still an important source of non-point source pollution and agricultural carbon emissions, inhibits the growth of OBTC [5,49].

With quadratic terms introduced, the variable intensity of environmental regulation (ER) shows a relatively complex association with the effect of biased technical change. The ER variable exerts a positive “U” effect on both IBTC and OBTC achieved in the eastern region (“U” inflection point is 0.01997 and 0.02002, respectively). The impact of the ER variable on IBTC and OBTC in the western region is positive and inverted “U”-shaped (“U” inflection point is 0.01714), respectively. According to the current regional average level of environmental regulation intensity, the western region is in the rising interval. By contrast, the eastern region is on the left side of the “U” inflection point, which is in the falling interval. This means that the current environmental regulation inhibits the growth of IBTC in eastern region to some extent, but it could play a positive role beyond the inflection point. Considering the regression results of environmental regulation on IBTC, it can be found out that the “compliance costs theory” applies to the impact of current environmental regulation on biased technological progress in the eastern region [41]. Since agricultural production is in a period of green transformation, the increase in pollution control costs will produce a crowding-out effect on the development and application of green technologies, and the production technology with bad output will still bring great benefits. However, this problem can be alleviated by further enhancing environmental regulation (crossing the “U” inflection point) to enter the “innovation and compensation” stage [42]. The practicalities of resource endowment and agricultural production in the western region are starkly different than in the eastern region, and the western region is less polluted. Currently, environmental regulation in the western region has a positive impact on both IBTC and OBTC, but the growth of OBTC will be inhibited when it is further improved (crossing the “U” inflection point).

## 6. Conclusions and Policy Implications

Based on the DEA-SBM model considering undesired output, this paper decomposes the agricultural green total factor productivity index (ML) to obtain the green-biased technical change (BTC) index in Chinese agriculture. Then, this paper selects agricultural carbon emissions and non-point source pollution into undesired output simultaneously, so as to calculate and identify the bias and characteristics of agricultural green technology change made in 29 Chinese provinces (including autonomous regions and municipalities) from 1998 to 2017. Furthermore, the impact mechanism of green-biased technical change is explored. The main conclusions of this study are presented as follows:

(1) During the research period from 1998 to 2017, technical change is the key driving force for improving China’s agricultural green total factor productivity, and green-biased technical change is crucial to maintaining the long-term and stable improvement of technical change. In contrast, the IBTC index plays a significant role in promoting technological progress, whereas the OBTC index impedes technological progress to some degree. According to the results of regional comparison, the BTC index in the eastern region shows a trend of “one high and one low”. Specifically, among all regions, the eastern region has the highest IBTC index, whereas its OBTC index is the lowest.
(2) According to the identification results about the characteristics of green-biased technical change, China’s agricultural production shows a clear tendency of capital substituting for labor and land during the period from 1998 to 2017. On the one hand, it promotes the growth of IBTC, thus enhancing the improvement of agricultural GTFP. On the other hand, the application of agricultural technology with capital support continues to cause severe environmental problems. In general, the substitution effect of desirable output on undesirable output at the national level is slightly stronger and the advantage is not immediately apparent, but there has been a positive trend over the recent few years. The results of the regional comparison show that the eastern region has the most severe environmental problems with agricultural production.
(3) As indicated by the regional grouping regression results obtained for the influencing factors of green-biased technical change, IBTC is promoted by the degree of marketization, the level of urbanization, the degree of capital deepening, and the intensity of financial support for agriculture. By contrast, the OBTC is mostly inhibited by these variables. As for the proportion of the farming sector and animal husbandry, it only has a positive impact on the growth of IBTC in the central region. Differently, the proportion of animal husbandry exerts a negative effect on OBTC to some extent. There is regional heterogeneity observed in the impact of environmental regulation intensity on biased technical change. In the eastern region, there is a period of transition from the “compliance costs” stage to the “innovation compensation” stage, whereas in the western region, there is a positive impact on biased technical change, which is due to its special resource endowment and economic development characteristics. However, a further enhancement of environmental regulation in the western region will make it counterproductive.

The above conclusions have significant implications for the guidance on the agricultural greening transformation and development in China. The main points are stated as follows:

(1) There is a close correlation between the biased changes in agricultural technology progress and the factor endowment structure of agricultural development in different periods. As an important source of agricultural growth in China’s transition period, the substitution of agricultural capital for labor and land factors represents an inevitable choice to ensure the continued balance between the supply and demand of agricultural products, given the current conflict between people and land and the development of a new period of urbanization. For compliance with this change in the mode of agricultural production, it is necessary to deepen the market-oriented reform of factors while giving full play to the market mechanism, and to provide an institutional guarantee for the deepening of agricultural capital, the transfer of agricultural labor force, and the orderly circulation and large-scale use of land.
(2) The biased technical change of agriculture should be evaluated differently based on the circumstances of various provinces. Regions should seize the opportunities presented by the changes in factor endowments during the transformation of agriculture, maximizing the use of the regional advantages’ resource endowments and adapting their resources to local conditions in order to optimize the allocation and combination of capital, labor, and land. Through the optimization of industrial structure, operations on a moderate size and related facilities are enhanced, the regional comparative advantage is exerted, and the biased technical progress is steered in accordance with the regional agricultural production mode.
(3) The development of agricultural green technology should be actively guided toward the stage of “innovation compensation” by implementing effective strategies of integrated innovation and demonstration promotion; for example: strengthening the guiding role of financial, taxation, fiscal, and other preferential means in the investment of agricultural enterprises in green technology R&D development; establishing a mechanism for the in-depth integration of industry, university, and research institutes, strengthening green-oriented technology research in agriculture, and bringing into play the synergistic innovation effect; stimulating the transformation and application of green technology achievements in agriculture by improving a multi-body and multi-dimensional agricultural science and technology promotion network, as well as pilot demonstrations in agricultural green development pioneer areas.
(4) Reasonable environmental regulations may effectively minimize pollution emissions in the production process, conserve the input of production factors, and hasten the transformation of agricultural technology progress towards energy conservation and emission reduction. On the one hand, local governments should control the intensity of environmental regulations and properly balance economic performance and environmental performance based on the phased characteristics of regional agricultural development. On the basis of the comprehensive treatment of agricultural pollution, the reduction of agricultural inputs and their scientific use should be further promoted, and the resource utilization of livestock and poultry manure should be strengthened. On the other side, the government should energize the market-driven environmental regulations and build and enhance ecological compensation methods such as resource use rights, emissions trading, and carbon emissions trading.
(5) Lastly, from the perspective of strategic foresight, with the rapid development of digitalization and intelligence and their deep penetration into agriculture, as well as other industries, the trend of capital substituting for labor and land will become increasingly apparent, having a revolutionary effect on the development of agricultural green technology. To promote the high-quality development of agriculture, it is essential to seize this period of strategic opportunity and layout in advance, and effectively implement the effective agglomeration and optimal allocation of agricultural capital, land, labor, and so on.

## Figures and Tables

**Table 1:** Identification method of green technical change bias.

**Input**	**IBTC > 1**	**IBTC = 1**	**IBTC < 1**
$\frac{I^{t+1}}{J^{t+1}}/\frac{I^{t}}{J^{t}}>1$	Technological progress of I using and J saving	Neutral	Technological progress of J using and I saving
$\frac{I^{t+1}}{J^{t+1}}/\frac{I^{t}}{J^{t}}<1$	Technological progress of J using and I saving	Neutral	Technological progress of I using and J saving
**Output**	**OBTC > 1**	**OBTC = 1**	**OBTC < 1**
$\frac{Y^{t+1}}{B^{t+1}}/\frac{Y^{t}}{B^{t}}>1$	Environmentally friendly technological progress	Neutral	Environmental degradation technological progress
$\frac{Y^{t+1}}{B^{t+1}}/\frac{Y^{t}}{B^{t}}<1$	Environmental degradation technological progress	Neutral	Environmentally friendly technological progress

**Table 2:** Agricultural carbon emission.

Source	Sub Source	Reference Sources for Carbon Emission Coefficient
Agricultural materials	Chemical fertilizers (including nitrogen, phosphorus, potassium and compound fertilizers), pesticides, and agricultural films	Zhang et al. (2019), West (2002), Cheng et al. (2011) [63,64,65]
Rice cultivation	Early rice, middle rice, and late rice	Min et al. (2012), Tian et al. (2022) [5,61]
Livestock breeding *	Cattle (including beef cattle, dairy cattle, buffalo), sheep (including goats and sheep), pigs, poultry	Min et al. (2012), Huang et al. (2022) [6,61]
Agricultural energy	Agricultural diesel oil	IPCC

Note: * Taking into account the growth cycle of livestock and the poultry breeding process, the breeding period of cattle and sheep is more than 1 year, and the total amount of breeding is measured by the end of the inventory; as the breeding period of pigs and poultry is less than 1 year, the total amount of breeding is measured by the amount slaughtered in that year (the same with the ANSP estimation below).

**Table 3:** Agricultural non-point source pollution unit table.

Activity	Class	Unit	Indicator	Discharged Pollutants
Fertilizer runoff	Nitrogenous Fertilizer (NF)	NF use for grain crops NF use for vegetables NF use for other crops	NF consumption (10^4^ t)	TN, TP
	Phosphate Fertilizer (PF)	PF use for grain crops PF use for vegetables PF use for other crops	PF consumption (10^4^ t)	
	Compound Fertilizer (CF)	CF use for grain crops CF use for vegetables CF use for other crops	CF consumption (10^4^ t)	
Livestock and poultry breeding *	Livestock	Cow and cattle	Year-end inventory (10^4^ head)	CODcr, TN, TP
		Pig	Slaughtered number (10^4^ head)	
		Sheep	Year-end inventory (10^4^ head)	
	poultry	Poultry	Slaughtered number (10^4^ head)	
Agricultural organic waste	Grain crops	Rice Wheat Beans Corn	Yield (10^4^ t)	CODcr, TN, TP
	Economic crops	Oil-bearing crops Vegetables, fruits	Yield (10^4^ t)	
Rural sewage Rural wastes	Rural wastewater Rural solid waste	Person	Rural population (10^4^ person)	CODcr, TN, TP
		Person	Rural population (10^4^ person)	

Note: * Taking into account the growth cycle of livestock and the poultry breeding process (the same with carbon emission estimation above).

**Table 4:** Measurement results of BTC index decomposition of each region.

Year	OBTC			IBTC		
	Eastern Region	Central Region	Western Region	Eastern Region	Central Region	Western Region
1998–2000	0.98259	0.99587	1.00060	1.00291	1.00066	1.00140
2001–2005	1.00251	0.99583	0.98209	1.00873	1.00087	1.00107
2006–2010	0.99784	0.99993	0.99981	1.01579	0.99761	1.00066
2011–2015	0.99211	0.99994	1.00321	1.01090	0.99871	0.99995
2016–2017	0.99865	1.00040	1.00165	1.01396	1.00187	1.00280
Average	0.99535	0.99834	0.99650	1.01068	0.99958	1.00091

**Table 5:** Input bias and output bias statistics of each region.

Region	Capital and Labor		Capital and Land		Land and Labor		Desirable and Undesirable Output	
	Capital	Labor	Capital	Land	Land	Labor	Desirable Output	Undesirable Output
Eastern Region	164	56	156	64	110	110	90	130
Central Region	120	40	121	39	92	68	104	56
Western Region	161	39	160	40	123	77	129	69
Total samples	445	135	437	143	325	255	323	255

Note: Since the OBTC index of Sichuan in 1999 and Ningxia in 2014 is 1, these two data are missing in the output bias statistics (the same below).

**Table 6:** Descriptive statistics.

	Mean	Standard Dev	Minimum	Maximum	Skewness	Kurtosis	Jarque–Bera Test	Observations
lnIBTC	0.0038	0.0173	−0.00448	0.1035	2.9596	17.7605	6112 ***	580
lnOBTC	−0.0029	0.0194	−0.1116	0.0510	−2.7606	16.4924	5136 ***	580
lnMA	1.8080	0.3505	0.8078	2.3921	−0.4792	2.6509	25.15 ***	580
lnUL	−0.7793	0.3308	−1.6245	−0.1187	−0.2409	2.7983	6.593 **	580
lnCD	−1.2450	0.7378	−4.3559	−0.0721	−1.8913	8.3489	1037 ***	580
lnFS	−2.3735	0.4069	−3.7297	−1.7259	−1.0571	4.0012	132.3 ***	580
ER	0.0128	0.0072	0.0029	0.0403	1.5223	5.5945	386.7 ***	580
ER^2^	0.0002	0.0003	8.41 × 10^−6^	0.0016	2.8749	12.2450	2865 ***	580
lnDR	−1.5735	0.7650	−6.2146	−0.3368	−1.3051	6.4919	453.8 ***	573
lnPF	−0.6497	0.1673	−1.0023	−0.3011	0.1179	2.4222	9.412 ***	580
lnAH	−1.2168	0.2899	−1.8643	−0.6143	−0.0849	2.5101	6.499 **	580

Note: ** *p* < 0.05, *** *p* < 0.01.

**Table 7:** Correlation matrix.

	lnIBTC	lnOBTC	lnMA	lnUL	lnCD	lnFS	ER	ER^2^	lnDR	lnPF	lnAH
lnIBTC	1.0000										
lnOBTC	/	1.0000									
lnMA	0.1968 ***	−0.0293	1.0000								
lnUL	0.1972 ***	−0.0667	0.5677 ***	1.0000							
lnCD	0.1386 ***	−0.1056 **	0.3600 ***	0.3896 ***	1.0000						
lnFS	−0.1717 ***	0.1138 ***	−0.3419 ***	−0.1935 ***	−0.3379 ***	1.0000					
ER	−0.0294	−0.0767 *	−0.1062 **	0.1811 ***	0.2118 ***	0.2150 ***	1.0000				
ER^2^	0.0068	0.0068 **	−0.2106 ***	0.0314	0.0927 **	0.1328 ***	0.7102 ***	1.0000			
lnDR	−0.1090 ***	−0.0255	−0.4871 ***	−0.2726 ***	−0.1114 ***	0.1850 ***	0.0831 **	0.0945 **	1.0000		
lnPF	−0.1444 ***	0.0941 **	−0.4583 ***	−0.2931 ***	−0.2013 ***	0.3939 ***	0.1514 ***	0.1651 ***	0.1151 ***	1.0000	
lnAH	−0.1063 **	−0.0005	−0.1940 ***	−0.0418	−0.0507	−0.0616	0.0915 **	0.0688 *	0.1643 ***	−0.2792 ***	1.0000

Note: * *p* < 0.10, ** *p* < 0.05, *** *p* < 0.01.

**Table 8:** Coefficient covariance matrix.

	lnIBTC	lnOBTC	lnMA	lnUL	lnFS	ER	ER^2^	lnDR	lnPF	lnAH
lnIBTC	0.0003									
lnOBTC	/	0.0004								
lnMA	0.0011	−0.0003	0.1213							
lnUL	0.0011	−0.0006	0.0628	0.1055						
lnCD	0.0017	−0.0015	0.0921	0.0937						
lnFS	−0.0011	0.0008	−0.0456	−0.0225	0.1571					
ER	−0.0004	−0.0012	0.0329	0.0672	0.0856	1.0025				
ER^2^	−0.0003	−0.0035	−0.1581	0.0252	0.1147	1.5377	4.6386			
lnDR	−0.0014	−0.0003	−0.1298	−0.0677	0.0561	0.0636	0.1576	0.5853		
lnPF	−0.0004	0.0002	−0.0271	−0.0164	0.0265	0.0257	0.0571	0.0148	0.0282	
lnAH	−0.0005	0.0001	−0.0184	−0.0019	−0.0087	0.0243	0.0099	0.0364	−0.0136	0.0840

**Table 9:** Unit root test.

Variables	LLC Test	ADF-Fisher Test	PP-Fisher Test
LnIBTC	−18.2225 ***	145.8261 ***	429.1882 ***
LnOBTC	−22.0813 ***	292.1797 ***	620.4112 ***
lnMA	−10.7147 ***	148.6061 ***	289.9698 ***
lnUL	−31.8652 ***	381.2459 ***	87.7849 ***
lnCD	−1.2960 *	107.0598 ***	78.1833 **
lnFS	−5.7594 ***	492.2177 ***	125.3569 ***
ER	−10.0466 ***	135.7645 ***	218.8106 ***
lnDR	−11.8420 ***	132.1506 ***	383.0298 ***
lnPF	−4.2352 ***	83.1229 **	75.0570 *
lnAH	−3.3904 ***	107.2212 ***	76.3836 *

Note: * *p* < 0.10, ** *p* < 0.05, *** *p* < 0.01.

**Table 10:** Summary of F test, Breusch–Pagan LM test, and Hausman test results.

Explained Variable	Test	Statistics	*p* Value
lnIBTC	F test	3.02 ***	0.0000
	Breusch–Pagan LM test	25.18 ***	0.0000
	Hausman test (Cross-section Random)	21.539 **	0.0105
lnOBTC	F test	1.64 **	0.0059
	Breusch–Pagan LM test	2.66 *	0.0513
	Hausman test (Cross-section Random)	16.370 *	0.0595

Note: * *p* < 0.10, ** *p* < 0.05, *** *p* < 0.01.

**Table 11:** Regression analysis results of influencing factors on green-biased technical change in Chinese agriculture.

Variables	lnIBTC				lnOBTC			
	(1)	(2)	(3)	(4)	(5)	(6)	(7)	(8)
	Total Samples	Eastern Region	Central Region	Western Region	Total Samples	Eastern Region	Central Region	Western Region
lnMA	0.0021 (0.0035)	0.0363 *** (0.0087)	0.0083 (0.0112)	−0.0001 (0.0033)	0.0018 (0.0088)	−0.0173 (0.0362)	0.0198 * (0.0101)	−0.0065 (0.0072)
lnUL	0.0052 * (0.0027)	0.0027 (0.0077)	0.0053 (0.0044)	0.0042 ** (0.0014)	−0.0102 *** (0.0028)	−0.0272 *** (0.0054)	−0.0413 *** (0.0071)	−0.0010 (0.0010)
lnCD	−0.0025 (0.0017)	0.0117 ** (0.0052)	−0.0019 (0.0017)	−0.0013 (0.0009)	−0.0028 (0.0028)	−0.0300 *** (0.0058)	−0.0058 *** (0.0015)	0.0050 ** (0.0020)
lnFS	0.0100 *** (0.0025)	0.0229 * (0.0114)	0.0027 (0.0027)	−0.0003 (0.0022)	−0.0078 (0.0046)	−0.0061 (0.0138)	−0.0006 (0.0048)	−0.0082 (0.0062)
ER	−0.7761 *** (0.1574)	−2.0264 *** (0.4751)	−0.1211 (0.3218)	0.3311 * (0.1608)	−0.1151 (0.2135)	−1.8207 ** (0.7417)	0.8116 (0.8059)	0.9338 *** (0.1812)
ER^2^	12.7479 *** (4.4072)	50.7286 *** (16.6306)	−2.9618 (10.5899)	−6.6953 (3.9956)	−1.8625 (6.2291)	45.4718 *** (12.0672)	−15.2118 (16.2269)	−27.2374 *** (7.4569)
lnDR	0.0006 (0.0004)	0.0028 ** (0.0011)	0.0007 (0.0009)	0.0003 (0.0003)	−0.0027 *** (0.0008)	−0.0040 *** (0.0012)	−0.0006 (0.0009)	−0.0002 (0.0010)
lnPF	−0.0503 *** (0.0147)	−0.1131 *** (0.0243)	0.0499 * (0.0260)	−0.0098 (0.0104)	0.0144 (0.0121)	0.0924 * (0.0444)	−0.0420 (0.0266)	0.0078 (0.0049)
lnAH	−0.0147 * (0.0076)	−0.0358 *** (0.0094)	0.0231 *** (0.0079)	−0.0055 (0.0080)	−0.0177 *** (0.0049)	−0.0088 (0.0123)	−0.0095 (0.0081)	−0.0070 ** (0.0032)
Constant	−0.0084 (0.0128)	−0.0702 * (0.0395)	0.0000 (0.0000)	0.0000 (0.0000)	−0.0836 ** (0.0343)	−0.0385 (0.0800)	0.0000 (0.0000)	0.0000 (0.0000)
R^2^	0.2776	0.3426	0.2151	0.2671	0.1649	0.2386	0.3680	0.2790
F test	167.8366 ***	5028.2179 ***	76.3014 ***	205.0315 ***	179.2126 ***	1455.8758 ***	189.5409 ***	118.9316 ***
Sample	573	213	160	200	573	213	160	200
Year Effect	Yes	Yes	Yes	Yes	Yes	Yes	Yes	Yes
Region Effect	Yes	Yes	Yes	Yes	Yes	Yes	Yes	Yes

Note: Driscoll–Kraay Standard errors in parentheses, * *p* < 0.10, ** *p* < 0.05, *** *p* < 0.01.

## Data Availability

The data are not publicly available due to personal privacy and non-open access to the research program.
